# The Roles of Neutrophils in the Pathogenesis of Liver Diseases

**DOI:** 10.3389/fimmu.2021.625472

**Published:** 2021-03-08

**Authors:** Jiaojiao Tang, Zijun Yan, Qiyu Feng, Lexing Yu, Hongyang Wang

**Affiliations:** ^1^Division of Life Sciences and Medicine, Cancer Research Center, The First Affiliated Hospital of University of Science and Technology of China, Hefei, China; ^2^International Cooperation Laboratory on Signal Transduction, Ministry of Education Key Laboratory on Signaling Regulation and Targeting Therapy of Liver Cancer, Shanghai Key Laboratory of Hepato-Biliary Tumor Biology, Eastern Hepatobiliary Surgery Hospital, Shanghai, China; ^3^Graduate Management Unit, Shanghai Changhai Hospital, Second Military Medical University, Shanghai, China; ^4^National Center for Liver Cancer, Shanghai, China

**Keywords:** neutrophils, liver regeneration, alcoholic liver disease, non-aloholic liver disease, hepatocellular carcinoma

## Abstract

Neutrophils are the largest population of circulating leukocytes and the first responder against invading pathogens or other danger signals. Sophisticated machineries help them play critical roles in immunity and inflammation, including phagocytosis, superoxide production, cytokine and chemokine production, degranulation, and formation of neutrophil extracellular traps (NETs). After maturation and release from the bone marrow, neutrophils migrate to inflamed tissues in response to many stimuli. Increasing evidences indicate that neutrophils are critically involved in the pathogenesis of liver diseases, including liver cancer, thus making them promising target for the treatment of liver diseases. Here, we would like to provide the latest finding about the role of neutrophils in liver diseases and discuss the potentiality of neutrophils as target for liver diseases.

## Introduction

Neutrophils are the most abundant white blood cells in mammals, representing the first line of innate defense against invading pathogens or other foreign bodies. Moreover, they play significant roles in shaping adaptive immunity and function as coordinators of the overall immune and inflammatory responses. Sophisticated processes, including phagocytosis, reactive oxygen species (ROS) generation, degranulation, cytokines and chemokines production, and neutrophil extracellular traps (NETs) release are vital for the immunological functions of neutrophils (1). Neutrophil loss or deficiency due to diseases or side effects of therapy is usually associated with severe recurrent infection (2, 3). However, excess infiltration and/or activation of neutrophils in the tissue can cause chronic inflammation, limit tissue repair, and lead to loss of organ function (4). Previous studies have indicated that neutrophil-induced inflammation occurs during the pathogenesis of a range of chronic diseases and cancer. Therefore, neutrophils represent a promising therapeutic target for various diseases (1), and various targeting approaches, including targeting neutrophil development and production, interfering with neutrophil accumulation at the site of infection/inflammation and reversing the detrimental changes of neutrophil phenotype that occur during certain pathological conditions, as well as mitigating the harmful effects of NETs (1, 2) have emerged.

The role of neutrophils in the pathogenesis of liver diseases has garnered intense interest in recent years. Neutrophils routinely patrol the liver sinusoids and there are few resident neutrophils in the liver. Instead, they can be recruited into the liver rapidly during acute liver infection or injury and serve as the principal phagocyte type responsible for pathogen clearance. The infiltration of neutrophils is commonly seen in all types of liver diseases. However, the overwhelming activation of neutrophils can also induce liver damage. Therefore, neutrophils are considered to be double-edge swords during acute liver inflammation. The importance of neutrophils in the chronic liver diseases (CLD) has also been appreciated in recent years because they can communicate with other immune and non-immune cells within the liver. In the recent review, we would like to provide the emerging evidence for the relevance of neutrophils during various liver diseases, and discuss the potentiality of neutrophils as target for liver diseases. We also discuss how CLD affects granulopoiesis, neutrophil phenotype, and function.

## Production of Neutrophils and Mediators of Their Function

Since they are among the shortest-lived cells in mammals, neutrophils rely on constant replenishment from the bone marrow through highly controlled granulopoiesis (~10^11^ neutrophils are produced from the human body every day) (5), and further increase under stress conditions (which is called “emergency granulopiesis”) (6). They originate from haematopoietic stem cells to common myeloid progenitor to lineage-committed progenitors that mature into neutrophils (3). Transcription factors, such as CCAAT/enhancer binding protein (C/EBPα), PU 1, and RUNX1 are necessary for neutrophil maturation during steady-state granulopoiesis, while C/EBPβ severs as a master regulator for emergency granulopoiesis (6, 7). Neutrophil production, maturation, release, and elimination are under tight control to maintain homeostatic stability and balance between antimicrobial and proinflammatory functions. A major regulator is granulocyte colony-stimulating factor (G-CSF), which promotes neutrophil development by engaging with G-CSF receptor and their release via downregulation of CXCR4 and upregulation of CXCR2 in neutrophils (1). Release mature neutrophils then migrate into inflamed tissues in response to various stimuli, such as chemokines (CXCL1, CXCL2) gradient.

The maturation of neutrophils is characterized by the condensation and mutilobular appearance of the nucleus, and the emergence of cell type-specific intracellular granules (7). During granulopoiesis, three types of granules are formed consecutively, namely, primary, secondary, and tertiary, as well as secretary vesicles of endocytic origin, all of which are prepackaged with antimicrobial and tissue-destructive factor, along with various neutrophil receptors. They are all readily available to be released to participate in the host response to inflammation or infection (1, 8). For instance, the azurophil (primary) granules are the reservoirs of myeloperoxidase (MPO), neutrophil elastase (NE), proteinase 3, (PR3) and most proteolytic and bactericidal proteins, and are considered to be the microbicidal compartment mobilized during phagocytosis. The specific (secondary) granules harbor antimicrobial lactoferrin, neutrophil gelatinase-associated lipocalin (LCN2), and chitinase-3-like protein-1. Gelatinase (tertiary) granules contain matrix metalloproteinase 9 (MMP9), collagenase (MMP8), and cathelicidin antimicrobial peptide. Secretory vesicles are rich in transmembrane receptors that integrate into the plasma membrane as exocytosis occurs (9).

Another weapon that helps neutrophils to capture pathogens is NETs, which are extracellular structures composed of chromatin coated with histones, proteases, and granular and cytosolic proteins. The formation of NETs is complicated and has been reviewed elsewhere (3, 10). NETs bind viruses, bacteria, fungi, and parasites, preventing their spread. They can also trap platelets and erythrocytes to initiate coagulation, and trap tumor cells to promote their spread (11). Thus, inappropriately formed or improperly degraded NETs can become pathogenic and are implicated in various non-infectious diseases such as CLD and cancer.

## Neutrophils and Acute Liver Diseases

### Neutrophils and Two-Thirds Partial Hepatectomy

The liver has a remarkable regenerative capacity with compensatory re-growth of the liver after liver damage, including physical resection or chemical injury (12, 13). Liver regeneration is a complicated and well-organized process involving multiple genes and signaling pathways that initiate or promote liver regeneration. Most knowledge on liver regeneration comes from the rodent model of two-thirds partial hepatectomy (PHx). In this model, two-thirds of the rodent liver is removed surgically, and mature quiescent hepatocytes of the remnant liver proliferate to restore the original liver mass and function (14). Inflammatory cells such as Kupffer cells (KCs), dendritic cells, and T cells control this process either through direct interactions with hepatocytes or indirectly by releasing inflammatory cytokines (15). However, studies regarding the role of neutrophils in liver regeneration are limited. Neutrophils promote liver regeneration by binding intracellular adhesion molecule (ICAM-1), triggering KC-dependent release of hepatocyte mitogens, interleukin (IL)-6 and tumor necrosis factor (TNF) α (16). This is demonstrated in neutropenic mice, which show delayed liver regeneration and reduced hepatic levels of TNFα and IL-6 (16). Furthermore, significant changes in neutrophil phenotype are observed in patients who undergo PHx. This has been proposed to be important in defense against gut-derived endotoxins following hepatic resection (17, 18).

### Neutrophils and Drug/Chemical-Related Liver Injury

Drug/chemical-related liver injury [such as acetaminophen (APAP) and tetracarbon chloride (CCl_4_)] can result from chemical/drug-induced oxidant stress and tissue injury and/or by the local upregulation of inflammatory mediators (19), and is usually accompanied by a huge infiltration of neutrophils in the liver during the early phase (20). Danger-associated molecular patterns (DAMPs), such as HMGB1 and lipid peroxidation products from dying hepatocytes, and proinflammatory mediators such as IL-1β and TNFα released from KCs can guide neutrophils into damaged tissues, leading to a multistep process that involves ATP release, adhesion molecule upregulation, formation of a chemical gradient (CXCL1, CXCL2), formyl peptide signals, and finally clearance of necrotic debris (21, 22). Neutrophil invasion often aggravates the liver by the secretion of cytotoxic reactive oxygen and nitrogen species or proinflammatory cytokines such as IL-1β and TNF (22). In mouse models of acute and chronic liver injury, TLR2 and the S100A8–S100A9 signaling pathway act as key regulators of hepatic CXCL2 and TNF expression and subsequent neutrophil infiltration (23).

## Neutrophils and Liver Ischemia-Reperfusion

Severe liver damage may occur in ischemia-reperfusion (IR) during liver transplantation or surgical liver tumor resection, when the blood supply is restored after a long period of ischemia. This hepatic inflammation is initiated by the ischemic period but occurs mainly during the reperfusion phase and is characterized by a large neutrophil recruitment to the liver (24). In liver IR (LIR), the acute inflammatory response has two consecutive stages: ROS exacerbates liver damage in the first 6 h of reperfusion, while neutrophil recruitment plays a major role in the next 18 h of reperfusion (25). The neutrophil-derived MMP9 can also promote the recruitment of neutrophils to the damaged site (24). The recruited neutrophils partially mediate damage through oxidative stress in a mitogen-activated protein kinase-activating protein kinase 2 (MK2)-dependent manner and the production of MPO (26, 27). And the neutrophils-derived MMP9 can promote recruitment and MPO activation of neutrophils (28), which form a positive loop to exacerbate the IR injury (IRI). COX-2 derived from hepatocytes reduces liver injury by decreasing endoplasmic reticulum stress, neutrophil infiltration, and oxidative stress, while escalating autophagy, and apoptosis (29). Similar to this mechanism, extracellular vesicles derived from human umbilical cord blood mesenchymal stem cells also moderate IRI by downregulating neutrophil respiratory burst and oxidative stress (30).

NE may have the ability to mediate adhesion and extravasation of neutrophils in IRI (31). In fact, the recruited neutrophils induce the production of macrophages monocyte chemoattractant protein-1 (MCP-1) through NE and oxygen-free radicals (32). MCP-1 upregulates the expression of ICAM-1 in endothelial cells and promotes the adhesion of neutrophils and endothelial cell damage (33). In addition to mediating endothelial cell damage and aggravating IRI, NE also aggravates IRI in other ways. In IRI, NE downregulate the expression of prostacyclin, which decreases the expression of downstream insulin-like growth factor 1, which has been reported to inhibit the expression of endothelial monocyte-activating polypeptide-II, a neutrophil chemotactic factor (34). Elevated NE, as the putative ligand of TLR4, causes the upregulation of TLR4 in macrophages and hepatocytes, which induces the inflammatory cascade responses in IR (35). NE inhibitor sivelestat treatment inhibits the infiltration and activation of neutrophils and apoptosis and reduces proinflammatory factors such as TNF-α and IL-6, and downregulates chemokines (36).

Net also mediates the inflammation, thrombotic diseases, cancer, and autoimmune diseases (37). LSECs/IL-33/ST2 axis (38), IL-17A (39), mast cell degranulation (40), and TIMP-1 (41) are the driving force of NET in LIR. NET has cytotoxic effects on hepatocytes *in vivo* and *in vitro*, and triggers a KC inflammation response by upregulating the inflammatory factors IL-1β, IL-6, TNF-α, and chemokines CXCL10 and MCP-1 (42). In addition, acrolein produced under chronic stress boosts oxidative burst and NET formation, which induces HepG2 nuclear and mitochondrial damage in IRI (43). Extravasated neutrophils cause hypochlorous acid (HOCl) to diffuse into hepatocytes and contribute to oxidative modification of proteins during the reperfusion phase (44). Neutrophils also damages hepatocytes by releasing proteases, TNF-α, TGF-β and leukotrienes (45). In turn, the histones and HMGB1, acting as DAMPs, from damaged hepatocytes also elicit NET formation by activating neutrophil TLR4- and TLR9-Myd88 signaling in LIR (42). The results indicate that a positive feedback loop is formed between NET formation and hepatocyte apoptosis, which mediates liver toxicity and organ injury. Therefore, targeting the associated mediator of neutrophils may be a useful way to improve the survival of patients after liver tranplantation or surgical liver tumor resection (Table 1). The other functions of neutrophils in liver IRI are shown in Tables 2, 3.

**Table 1:** Therapeutic targets of neutrophils in liver diseases.

**Neutrophils in liver diseases pathology**	**Disease examples**	**Therapeutic way**	**Therapeutic targets**	**References**
Insufficient function of neutrophils	HCC, ALD	Enhancement the function of neutrophils	Adding G-CSF	(46, 47)
Excessive function of neutrophils	LIR, APAP, NAFLD, acute and chronic liver injury (CCl4), ALD, HCC	Inhibition of neutrophils function	Targeting neutrophils NETs, blocking the signal transduction, targeting NE, targeting CXCR2-FPR1, inhibiting neutrophils recruitment, adding GR-1 antibody	(10, 22–24, 35, 36, 48–51)
Abnormal and pathogenic function of neutrophils	Acute and chronic liver injury, HCC	Restore the neutrophils function	Inhibiting *CCRK* or hepatic IL-6	(52, 53)

*G-CSF, granulocyte colony-stimulating factor; FPR1, formyl peptide receptor 1; CCRK, cell cycle-related kinase*.

**Table 2:** The function of neutrophils granule component in liver disease.

**Factors**	**Models of liver disease**	**Pathogenesis**	**References**
The granule components of neutrophils			
MPO	IRI	Oxidative damage to the tissue	(27)
	NAFLD	Modulate the infiltration of neutrophils and T cells, induce pro-inflammatory factors Increase liver cholesterol Promote NAFLD toward advanced stages with fibrosis	(54)
	ALD	Act as a marker for the infiltration of neutrophils, and predict the prognosis in patients with alcoholic cirrhosis	(55)
	Fibrosis	Activate HSCs, upregulate fibrosis-related genes, and induce the oxidative stress *in vitro* Induce the hepatocyte death *in vivo*	(56)
	HCC	Expedite the HCV infection to HCC	(57)
NE	IRI	Adherence and extravasation of leukocyte via basement membrane degradation Stimulates the production of MCP-1 by macrophages *in vitro* Decreases endothelial production of prostacyclin and insulin-like growth factor-I in rats	(31, 32, 58, 59)
	NAFLD	Insulin resistance Induces the activation of pro-inflammatory factors	(49, 50)
	ALD	Induces proteolytic damage	(60)
	Fibrosis	Induces proteolytic tissue damage	(61)
	HCC	Induces proteolytic damage	(62)
MMP9	IRI	Promotes recruitment and MPO activation of neutrophils	(28)
	NAFLD	Elevated MMP9 drives the NASH and fibrosis progress	(63)
	ALD	Regulates homeostasis of the liver microenvironment	(64)
	Fibrosis	Degrades ECM and basement membrane components	(65)
	HCC	Decreases cell apoptosis and promote tumor metastasis Acts as a strong angiogenic stimulant	(66, 67)

**Table 3:** The other activity of neutrophils in liver disease.

**Factors**	**Models of liver disease**	**Pathogenesis**	**References**
The other activity of neutrophils			
NET	IRI	Have cytotoxic effect on liver cells and trigger Kupffer cells inflammation response Trigger nuclear and mitochondrial damage	(38, 39, 41–43)
	NAFLD	Accelerate the establishment of a pro-inflammatory environment in NASH	(68)
	ALD	Related to sepsis inflammation levels	(69)
	Fibrosis	Promote hepatic inflammation and fibrosis	(70)
	HCC	Cytotoxic resistance Express inflammatory mediator from captured HCC Promote tumor invasion, angiogenesis, and growth	(68, 71)
Oxidative stress	IRI	Oxidative stress	(26, 29, 30)
	NAFLD	Aggravate tumor risk by reducing damage recognition and nucleotide resection repair	(72)
	ALD	Promote the transition from ALD to liver fibrosis	(73)
	Fibrosis	Upregulate of collagen synthesis in HSCs	(74)
	HCC	Have toxic effects on HCC	(75)

## Neutrophils and Chronic Liver Inflammation

### Liver Fibrosis

Liver fibrosis is the main consequence of chronic liver injury of any etiology and may progress to cirrhosis and liver cancer. Activation of hepatic stellate cells (HSCs) that transdifferentiate from vitamin A–storing pericyte-like cells to α-SMA-positive, collagen-producing myofibroblasts is now well-established as the central driver of fibrosis (76, 77).

Infiltration of neutrophils is commonly observed in patients as well as in mice with alcohol or non-alcohol-induced steatohepatitis (78, 79). However, the role of neutrophils during liver fibrogenesis remains controversial. On one hand, increased expression of neutrophil (and mast cells)-derived IL-17 is a common signature of advanced liver fibrosis, which upregulates the expression of TGF-β receptor in HSCs and promotes liver fibrosis, and blocking IL-17/IL-22 alleviates liver fibrosis (80). IL-17A (secreted by Vγ_2_T or T_h_17 T cells) also promotes the recruitment of neutrophils into the liver and promotes liver fibrosis induced by *Schistosoma japonicum* infection (81) or bile duct ligation (BDL) (82). Mechanistically, neutrophils are shown to activate HSCs via the production of ROS and MPO (56, 74, 83). Activated HSCs produce GM-CSF and IL-15 to promote neutrophil survival (83), and cytokine-induced neutrophil chemoattractant to facilitate the recruitment of neutrophils (84), thus creating a positive feedback loop and exacerbating liver fibrosis. Moreover, neutrophils downregulate the butyrate receptor GPR43 and upregulate the secretion of TNF-α and IL-6, thereby promoting intestinal microbial translocation and exacerbating CCl_4_-induced liver fibrosis (85). Overexpression of HNP-1, a type of α-defensin, promotes the proliferation and activation of HSCs (86). And neutrophils induce proteolytic tissue damage by NE (61).

On the other hand, neutrophils have also been shown to contribute to collagen degradation during the resolution of fibrosis via their expression of MMPs (65). A recent report also demonstrated that neutrophils mediate the resolution of liver inflammation and fibrosis through microRNA (miR)-223 delivery to liver macrophages, favoring macrophage polarization toward a regenerative phenotype (87). Interestingly, another recent study by Yang et al. (88) also identified the beneficial effects of neutrophil-derived ROS on polarizing macrophages toward an alternative or reparative and anti-inflammatory phenotype in an APAP-induced liver injury model. In addition, the injection of autologous bone marrow-derived macrophages in mice during CCl4-mediated liver injury has been shown to lead to the recruitment of neutrophils into the liver, upregulation of MMPs, and anti-fibrotic effects (89).

What makes the thing complicated is that there are some reports showed that neutrophils were dispensable for establishing chronic inflammation and hepatic fibrosis. One report showed that neutrophils have minimal effects on BDL-induced liver fibrogenesis, as there is no significant difference in the production and deposition of collagen in the livers of anti-neutrophil antiserum treated mice or mice with neutrophil dysfunction due to transgenic expression of IL-8 (90). Another report showed that neutrophils are not essential to the hepatotoxin α-naphthylisothiocyanate-induced liver fibrosis, as there was comparable fibrosis between wild type and *CXCR2* (the key receptor for neutrophil recruitment) konckout mice (91). While infiltration of neutrophils is a common feature of human liver diseases, defective neutrophil recruitment does not impact chronic liver fibrosis (23).

In conclusion, these data reveal an elaborate role of neutrophils during liver fibrosis, reflecting their adoptive ability to a phenotype tightly regulated by the integration of signals derived from the microenvironment.

### Alcoholic Liver Disease

Alcoholic liver disease (ALD) is a spectrum of liver injury, ranging from hepatic steatosis to alcoholic hepatitis and cirrhosis (92), which is caused by excessive alcohol consumption. Chronic hepatocellular injury and death are intimately related to oxidative ethanol metabolism. A large number of neutrophils can be found in the liver of ALD patients, and markers of neutrophils (such as Ly6G, MPO, E-selectin) are upregulated (93). Moreover, neutrophils-derived MPO act as a marker for the infiltration of neutrophils, and predict the prognosis in patients with alcoholic cirrhosis (55). DAMPs, which are released following necrotic cell death, trigger macrophage and neutrophil activation, with senescence (via natural killer cells) and autophagy being the major regulators of liver inflammation (94). Factors that mediate hepatic infiltration of neutrophils include the CXCL1/CXCR2 axis (95), LCN2 (96), IL-33/ST2 (97), osteopontin (98), E-selectin (99), and activated type I natural killer T cells (100). Recruited neutrophils then release H_2_O_2_, NE (60), protease 3 (92), and proinflammatory factors (IL-8, TNF-α), or downregulate anti-inflammatory IL-10 to contribute to ALD (101). Therefore, neutrophils are a major contributor to the development of ALD, and targeting them may be a promising therapeutic strategy for ALD. Indeed, the blockade of inflammatory mediators involved in neutrophil infiltration or deletion of neutrophils ameliorates alcoholic liver injury in mouse models of early steatohepatitis (48).

However, excessive alcohol consumption frequently exerts negative effects on neutrophils, including granulopoiesis, and neutrophil release and function (46). Advanced ALD is also accompanied by granulicytopenia (102) and impairment of neutrophil function (103, 104). Infectious complications, including septic infections, occur in ~50% of ALD patients, which are the main cause of death in these individuals (104, 105). Therefore, neutrophil therapy in ALD patients requires special caution. Administration of G-CSF to increase neutrophil counts and improve their function, in adjunction to standard therapy, has been shown to substantially increase the survival of patients with either severe alcoholic hepatitis or alcoholic liver failure (46).

### Non-alcoholic Fatty Liver Disease

Non-alcoholic fatty liver disease (NAFLD) is the most common chronic liver disease, with a worldwide prevalence of 25% (106). It is an umbrella term that covers a continuum of liver conditions varying in severity of injury and resulting fibrosis: from hepatic steatosis alone (non-alcoholic fatty liver or NAFL) to a more serious condition with inflammation, hepatocyte damage, and pericellular fibrosis [non-alcoholic steatohepatitis [NASH]] (107). The presence of metabolic syndrome in an individual is the strongest risk factor for NAFLD. Its common pathologic drivers are the accumulation of toxic lipid species, which induce hepatocellular stress, injury, and death, leading to fibrogenesis and genomic instability that predispose individuals to cirrhosis and hepatocellular carcinoma (HCC) (107).

NASH is characterized by hepatic neutrophil infiltration (79). The ratio of NE to α1-antitrypsin (108), plasma concentration of PR3 and NE (109), neutrophil-to-lymphocyte ratio (110), serum levels of LCN2 (111), NETs (68), and MPO (112) significantly increase in patients with NAFLD. And elevated MMP9 from the neutrophils drives the NASH and fibrosis progress (63). Factors that mediate hepatic infiltration of neutrophils include activation of T_h_17 cells expressing IL-17A (113, 114), MPO (54), and gut-microbiome-derived DAMPs (115, 116).

Neutrophils then release a plethora of factors that play important roles in NAFLD. NE is an important regulator of insulin signaling, and depletion of NE results in enhanced insulin sensitivity, attenuated inflammation, and decreased liver damage in high-fat diet-fed mice (49, 50). Neutrophil PR3 also mediates insulin resistance in NAFLD (117). Neutrophil-derived serine proteases, namely NE, PR3, and cathepsin G, are important for the activation of pro-IL-1β/pro-IL-18, which are essential for NASH (118). The combined knockout of *Caspase-1* and *NE/PR3* genes in mice results in reduced inflammation and liver steatosis (118).

MPO, found in the primary granules of neutrophils, is released into both the phagolysosomes and the extracellular environment upon neutrophil activation. MPO catalyzes the formation of reactive oxygen intermediates, such as HOCl, a potent oxidant that interacts with superoxide anions to induce hepatocyte death (119, 120). HOCl can also damage DNA and inhibit DNA repair, thus leading to HCC development in NAFLD (72). MPO can also activate HSCs and promote fibrogenesis in methionine- and choline- deficient diet-induced NAFLD (56). In addition, MPO triggers the polarization of M2-type macrophages, which express high levels of TGF-β, MMP1, and MMP12, and promote fibrosis in NASH (49).

Neutrophil-derived NETs were found to promote the accumulation of macrophage in the liver, which then establish a favorable inflammatory microenvironment for HCC growth in an experimental NASH model (68). Interestingly, blocking NETs does not affect steatosis and free fatty acid accumulation but inhibits HCC development (68).

## Neutrophils and HCC

HCC is a common result of chronic liver disease. Its pathogenesis varies, with the main cause being chronic viral infection or inflammatory environment caused by a large leukocyte infiltration. The multiplying neutrophils in the liver accelerate tumor angiogenesis, epithelial-mesenchymal transition, and growth by producing MMP-9 (66), NET, and hepatocyte growth factor, thereby exacerbating HCC and metastasis (121). Thus, the infiltrating neutrophils may be pro- or anti-tumorigenic depending on the complex tumor niche.

**Figure 1:**
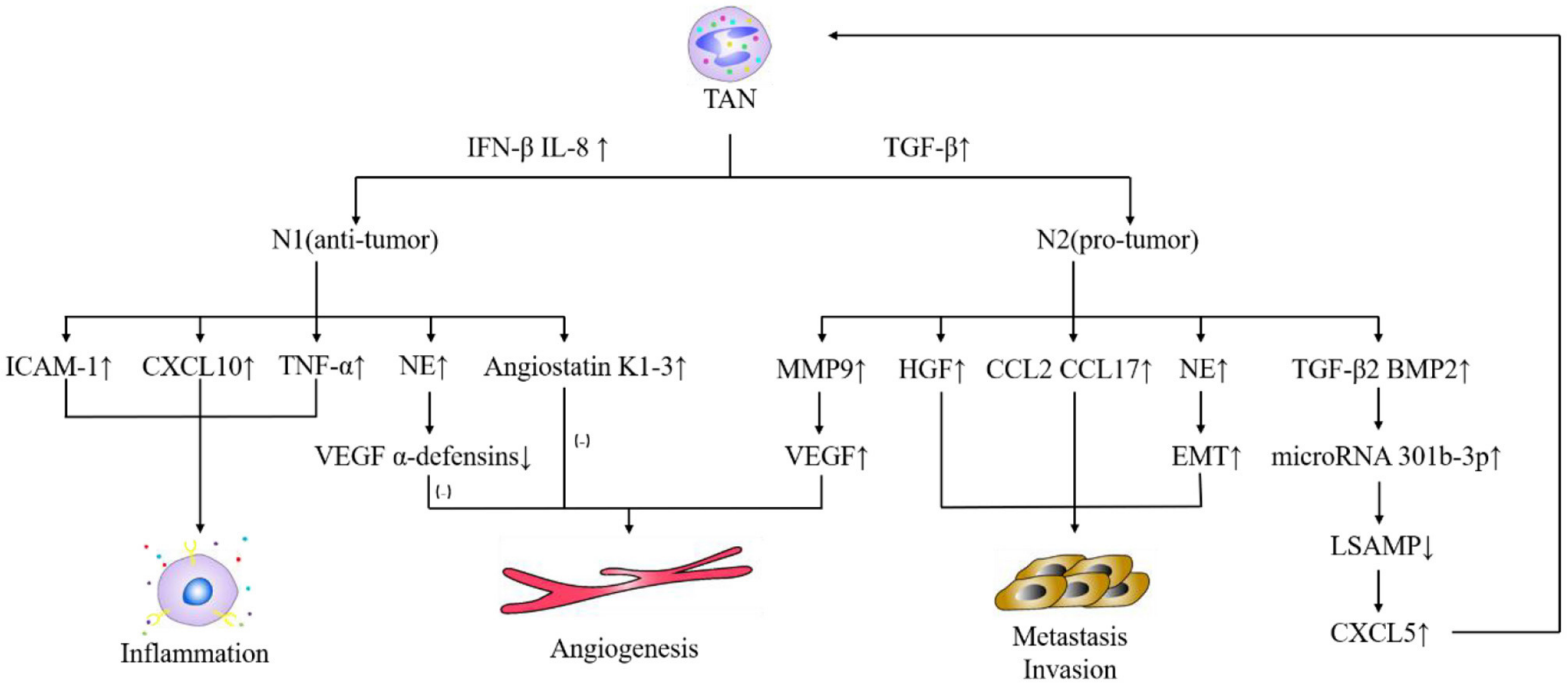
Dual roles of TAN in HCC. IFN-β, interferon-β; IL-8, interleukin-8; TGF-β, transforming growth factor-β; ICAM-1, intercellular cell adhesion molecule-1; CXCL, chemokine (C-X-C motif) ligand; TNF-α, tumor necrosis factor-α; NE, neutrophil elastase; VEGF, vascular endothelial growth factor; MMP9, matrix metalloproteinase 9; HGF, hepatocyte growth factor; CCL, chemokine (C-C motif) ligand; EMT, Epithelial-Mesenchymal Transitions; BMP2, bone morphogenetic protein 2; LSAMP, limbal gene expression membrane protein. TAN plays a critical role in pro-tumor and anti-tumor in HCC. Under the action of TGF-β (127), TAN differentiates into N2 phenotype, which promotes tumor development; while under the condition of IFN-β (128) and IL-8 (129), TAN differentiates into N1 phenotype, which limits tumor progression. N2 secretes MMP9, VEGF (128) to promote tumor angiogenesis; secretes HGF (130), CCL2, CCL17 (131), NE (132) to promote tumor metastasis and infiltration; secretes TGF-β2, BMP2 to promote the production of microRNA 301b-3p, inhibits the production of LSAMP, promotes the production of CXCL5. CXCL5 recruits more TAN (133). N1 secretes ICAM-1, CXCL10, TNF-α (124, 134) to promote inflammation. At this time, NE secreted by N1 will degrade VEGF α-defensins collaborating with Angiostatin K1-3 (123) secreted by N1 to inhibit angiogenesis and inhibit tumor progression.

In the tumor microenvironment, under the action of TGF-β, tumor-associated neutrophils (TAN) are divided into N1- and N2-TAN. TGF-β in the tumor microenvironment induces tumor-promoting N2-TAN, and blocks TGF-β-induced tumor-inhibiting N1-TAN (122). N1-TAN inhibits tumor progression through tumor cytotoxicity, inflammation, and immunity response. On one hand, the secreted elastase can promote the degradation of vascular endothelial growth factor A (VEGF-A), basic fibroblast growth factor, and α-defensin and inhibit angiogenesis (123). On the other hand, the secretion of ICAM-1, CXCL10, and TNF-α promotes inflammation and inhibits tumor growth (124). However, N2-TAN is more common in the tumor microenvironment. The greater inflammation caused by infiltrated TAN and tumor-associated macrophage is the primary causative factor for the high morbidity of liver cancer (125). TAN is directly involved in HCC *in vitro* and *vivo*. TAN releases TGF-β and bone morphogenetic protein 2, which upregulate miR301b-3p, which in turn is crucial for the formation of caner stem cells in HCC, which are characterized by low levels of E-cadherin and high levels of vimentin and N-cadherin (126). These cancer stem cells form a positive feedback loop with TAN (126). The dual roles of TAN in HCC are summarized in Figure 1. In addition, there are a group of circulating neutrophils, which have been shown to be a poor prognostic factor for overall survival of patients with HCC. They promote the development of HCC through p53 and STAT3 signaling pathways (135).

NET formation from neutrophils is higher in patients with HCC (71), and the high expression of NET supports HCC through cytotoxic resistance and an elevated inflammatory response (68). The interaction between captured HCC and internalization NETs contributes to the acquisition of invasive potential of HCC via TLR4/9-COX2 signaling (71). More importantly, the hypoxic environment within the tumor exacerbates NET formation, which forms a positive feedback loop to aggravate liver injury (136). Although DNase/PAD4^−/−^ mice do not exhibit changes in the progression of fatty liver, inhibiting the formation of NETs can successfully inhibit HCC growth (68). Recently, a clinical retrospective investigation revealed that higher pre-surgery NET levels are associated with shorter relapse-free survival/overall survival in patients with primary liver malignancies (137). This indicates that targeting NETs may be a potential therapeutic strategy against HCC.

Oxidative stress is critical for the development of HCC. Intracellular ROS and glutathione are elevated in neutrophils and others leukocytes in patients with early HCC (138). The neutrophils-derived oxidative stress partially initiates the HCC through MPO, which is expressed in neutrophils and KCs (139). MPO-derived HOCl damages DNA bases and impairs nucleotide excision repair, thus favoring somatic mutations and tumor progression (140). Activated neutrophils can also release cytochrome c via the production of ROS, which exerts anti-tumor effects against several carcinomas (141). The use of zinc oxide nanoparticles to mimic ROS from neutrophils or macrophages shows that ROS has toxic effects on HCC (75). The neutrophil-derived ROS against human HCC can be visualized at the cellular level (142).

Angiogenesis is critical for tumor progression, as blood provides oxygen and nutrients for cancer cells. The accumulation of neutrophils initiates the tumor angiogenic switch by releasing MMP9 in para-carcinoma from human HCC (51). MMP9 is produced by various types of cells, but human neutrophils can produce TIMP-free MMP9, which acts as a strong angiogenic stimulant (67). Elevated neutrophils can also upregulate the expression of VEGF and sinusoidal vasculature in HCC (51). Consistent with the above findings, neutrophils recruited in HCC and its products of IL-6 and IL-8 precipitate a proinflammatory microenvironment, which exacerbates the invasion of HCC *in vitro* and develops into angiogenesis and tumor metastasis *in vivo* (143). The inhibition of GR-1 with monoclonal antibodies has been shown to decrease tumor size and microvessel density in HCC-bearing mice (51).

In addition to affecting HCC itself, neutrophils affect the progression of HCC by acting on other immune cells. Cancer-associated fibroblasts in HCC enhance the level of programmed death-ligand 1 (PDL1)^+^ neutrophils via IL-6/STAT3, which is essential for the survival and functional activation of neutrophils (144). PD-L1^+^ neutrophils impair the function of T cells via PD-L1/programmed cell death protein 1 signaling (144). Neutrophils enhance the level of myeloid-derived suppressor cells, thereby inhibiting T cell function (145). Meanwhile, neutrophils also inhibit the interferon-γ production by peripheral blood mononuclear cells, which is associated with a low survival rate and high tumor burden (146), and can downregulate the IL-2-receptor-α and ICAM-1 receptor signaling, which in turn mediate cell-mediated immune resistance (147). This is indicated that targeting the neutrophils can improve the HCC by enhancing the activity of other immune cells.

## Conclusion

Hepatic infiltration of neutrophils is a common feature of most types of liver diseases. While they mainly function against invading pathogens and remove debris and dead cells, they can also induce and aggravate hepatocyte injury and promote the progression of liver diseases by producing ROS, degranulation, inflammation mediators, and/or shaping immunity. Therefore, neutrophils represent a potential target for liver diseases. Targeting strategies should be disease-specific, either to enhance, inhibit or restore neutrophil function, and some strategies have been in clinical use or in different stages of clinical trials (1, 2). However, it is of note that many models use anti-Ly6G antibodies to deplete neutrophils *in situ*, and debris of these died neutrophils in the injured tissue may exacerbate immune cell activation and phenotype, thus promoting liver diseases (148). New models are needed to overcome these potential drawbacks. The phenotypic and functional heterogeneity of neutrophils has been recognized in recent years (149); however, heterogeneity of neutrophils is largely unknown in liver diseases. A recent report revealed that neutrophils enter the tissues and acquires remarkable heterogeneity at the chromatin, RNA, and receptor levels (150). Therefore, new techniques such as single-cell sequencing, Assay for Transposase-Accessible Chromatin-sequencing (ATAC-seq), or multispectral imaging may help to thoroughly characterize the heterogeneity of neutrophils during the development of liver diseases, and provide new therapeutic approaches for the treatment of liver diseases.

## Author Contributions

JT drafted the manuscript. ZY and QF reviewed the manuscript structure and ideas. LY evaluated and reviewed manuscript structure, ideas, and science. HW conceived the topic and revised the manuscript. All authors listed have made a substantial, direct and intellectual contribution to the work, and approved it for publication.

## Conflict of Interest

The authors declare that the research was conducted in the absence of any commercial or financial relationships that could be construed as a potential conflict of interest.
